# Correlation of Necrotic Index and Modified Necrotic Index With the Harris Hip Score and Their Reliability in Patients With Osteonecrosis of the Femoral Head

**DOI:** 10.7759/cureus.82945

**Published:** 2025-04-24

**Authors:** Amit Saraf, Manan Gupta, Shahid Mir, Altaf Hussain, Krishna Kumar, Aneesh k Malhotra

**Affiliations:** 1 Orthopaedics, Teerthanker Mahaveer Medical College and Research Centre, Moradabad, IND; 2 Orthopaedics, Venkateshwara Institute of Medical Sciences, Gajraula, IND

**Keywords:** correlation mri, harris hip score, modified necrotic index, necrotic index, osteonecrosis of femoral head

## Abstract

Objective: This cross-sectional study aimed to evaluate the correlation between the necrotic index and the modified necrotic index with the Harris hip score (HHS), as well as to assess their interobserver and intraobserver reliability in patients with osteonecrosis of the femoral head (ONFH).

Methods: Fifty-four adult patients (88 hips) presenting with atraumatic hip pain and radiographic evidence of ONFH (Ficat-Arlet grades 1 and 2) were included. Participants abstained from analgesics for one week prior to the study. All underwent MRI, and HHS was calculated by a resident uninvolved in the study. The necrotic index was determined using angles obtained from mid-coronal and mid-sagittal MRI slices, whereas the modified necrotic index was derived from angles measured in MRI slices where the necrotic lesion appeared at its largest size. Three senior consultants performed measurements on two separate occasions to assess reliability.

Results: Both indices showed significant negative correlations with HHS. The modified necrotic index demonstrated a strong correlation (r = -0.940, p < 0.001), whereas the original necrotic index exhibited a moderate correlation (r = -0.790, p < 0.001). The interclass correlation coefficient (ICC) for assessment of lesion size was significant for both indices (p < 0.05), but it was higher for the modified index (ICC = 0.81) compared to the original index (ICC = 0.69). Levene’s test for homogeneity of variance indicated unequal variance across the two methods (p < 0.01). Measurement variance was smaller for the modified index than for the original index (p < 0.01), indicating greater precision. Both methods showed significant interobserver and intraobserver agreement (p < 0.05)

Conclusion: Both the necrotic index and the modified necrotic index showed significant negative correlations with HHS. The modified necrotic index demonstrated a strong correlation, whereas the original necrotic index exhibited a moderate correlation. Both methods proved reliable for measuring lesion size, with the modified method showing greater precision. Additionally, both indices demonstrated significant interobserver and intraobserver reliability.

## Introduction

Osteonecrosis of the femoral head (ONFH) is a debilitating condition caused by the disruption of the blood supply to the femoral head, with both traumatic and atraumatic etiologies. Traumatic causes include femoral head and neck fractures, acetabular fractures, and hip dislocations; atraumatic ONFH can be idiopathic or secondary to factors such as excessive steroid use, hemoglobinopathies, alcohol consumption, and autoimmune diseases [1,2]. Despite ongoing research, the exact pathogenesis of ONFH remains unclear. Patients typically present with pain in the early stages and, in advanced grades, experience difficulty performing daily activities such as walking, sitting cross-legged, squatting, and using stairs, significantly impacting their quality of life.

Radiography is the most commonly used imaging modality for diagnosis; however, early changes may not be visible on radiographs. The MRI is the most sensitive noninvasive modality, particularly for detecting early-stage ONFH. It is estimated that 30,000 new cases of ONFH are diagnosed annually in the US, though the prevalence data in India remain unknown [3].

In the pre-collapse stages, treatment is typically conservative, involving lifestyle adjustments and supportive measures such as analgesics. Surgical options include core decompression with or without bone grafting. In advanced cases, hip arthroplasty is often required [4]. Although osteoarthritis is the most common indication for hip arthroplasty in Western countries and the US, ONFH is reported to be the leading indication in Asian countries [3]. Treatment decisions are based on clinical symptoms and the degree of femoral head necrosis.

Several methods have been developed to evaluate the extent of necrosis and predict disease progression and outcomes. One of the earliest radiographic classification systems was proposed by Ficat et al. and consisted of four grades [5]. In 1974, Kerboul et al. [6] introduced an angular method for measuring lesion size and predicting collapse based on radiographic measurements. This method was later modified by Koo et al. [7], who used mid-sagittal and mid-coronal MRI sections to develop the necrotic index (the Koo and Kim method). Cherian et al. [8] further modified this index by measuring the extent of necrosis from MRI sections showing the largest lesions in the sagittal and coronal planes, thus introducing the modified necrotic index (the Cherian method). Although a volumetric assessment of lesions, as described by Steinberg et al. [9], provides a three-dimensional measurement of lesion size, it requires specialized 3D software and is technically challenging for routine use.

Clinically, the most widely used functional score for hip pathology is the Harris hip score (HHS), which evaluates pain, function, range of motion, and activity across 13 items. A higher score indicates better function [10]. It is generally understood that a rapidly declining HHS, a higher necrotic index, and a poor hip score are associated with an increased risk of collapse. However, the relationship between angular measurements and clinical scores remains poorly understood. Combining HHS with imaging findings may provide a more comprehensive assessment of disease progression. This study aims to evaluate the correlation between the necrotic index (Koo and Kim method) and the modified necrotic index (Cherian method) with the HHS, and assess their interobserver and intraobserver reliability.

## Materials and methods

This prospective cross-sectional study was conducted at a tertiary care center in Moradabad, India, from November 2022 to December 2024. It was approved by the Teerthanker Mahaveer University Institutional Ethics Committee (approval no. TMU/IEC/2021-22/61) prior to recruitment, and informed consent was taken from all participants. The study adhered to the principles of the Helsinki Declaration.

Inclusion and exclusion criteria

Adult patients presenting with atraumatic hip pain and radiographic findings suggestive of ONFH, Ficat-Arlet grade 1; normal or minor osteopenia and grade 2; sclerosis or cystic changes (focal sclerosis only and crescent sign without flattening of femoral head) were included. Participants were required to remain drug-free (of analgesics) for one week prior to undergoing the MRI. Patients with traumatic hip pain, inflammatory or infective pathologies, previous surgical treatment for ONFH, pathology in other joints, contraindications to MRI, or those unwilling to participate or provide consent were excluded.

Sample size

Because of the unknown prevalence of ONFH in India, a formal sample size calculation could not be performed. Instead, patients were included in a time-bound manner. The final analysis included 88 hips (54 patients).

Objectives

The primary objective was to measure the necrotic index (Koo and Kim method) and the modified necrotic index (Cherian method) by MRI and evaluate their correlation with HHS. The secondary objective was to assess the interobserver and intraobserver reliability of both indices.

Measurement of necrotic indices

**Figure 1:**
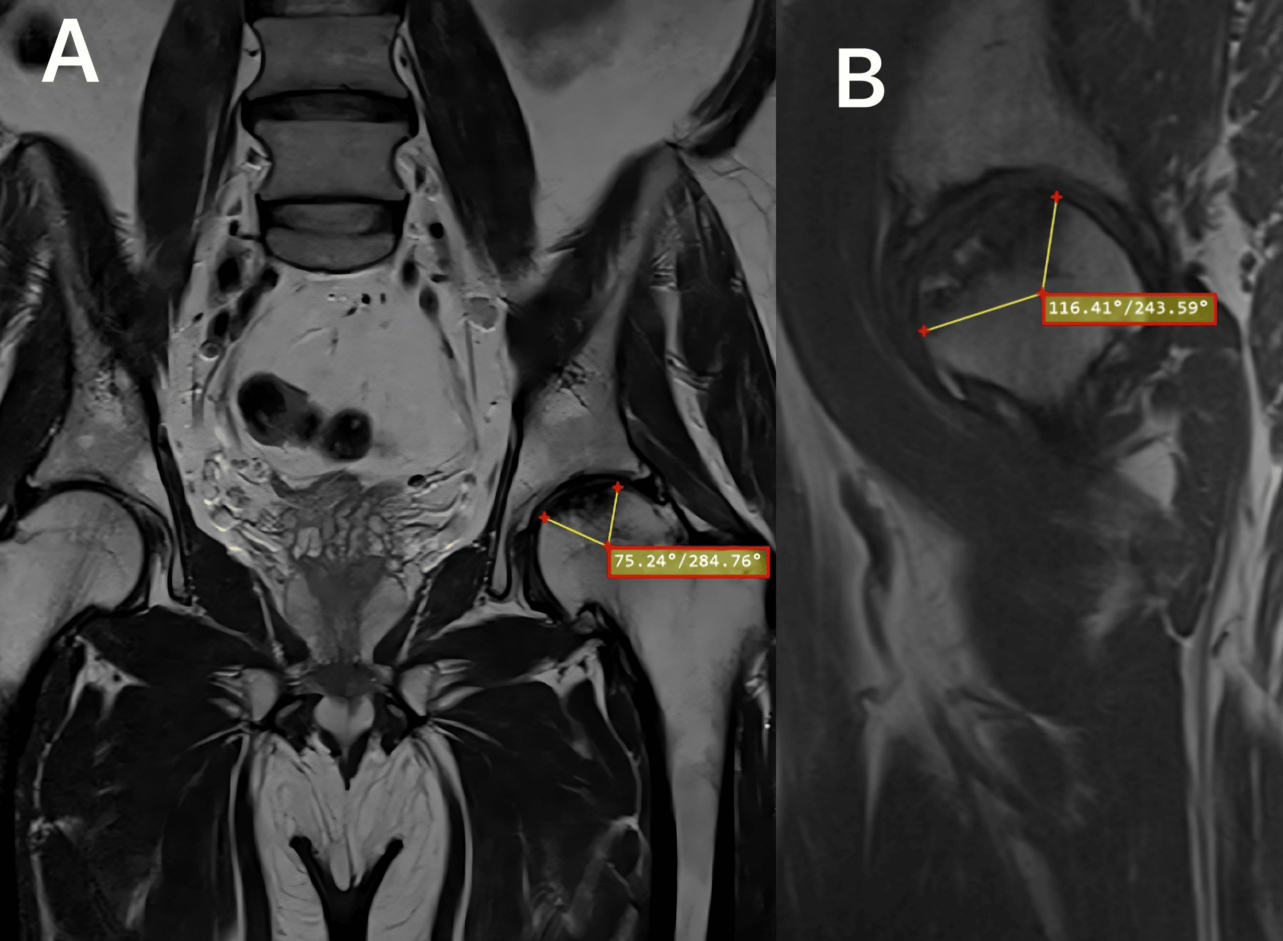
An example of the Koo and Kim method, which estimates lesion size using angles (yellow lines) measured on (A) mid-coronal and (B) mid-sagittal MRI images

The necrotic arc angle was measured in mid-sagittal (angle A) and midcoronal (angle B) MRI images. The percentage of necrosis (necrotic index) was calculated using the following formula as described by Koo et al. (Figure 1): necrotic index = (A/180) × (B/180) × 100 [7]. Based on these values, hips were classified into small (≤33%), medium (34% to 66%), and large (>66%) areas of necrosis.

**Figure 2:**
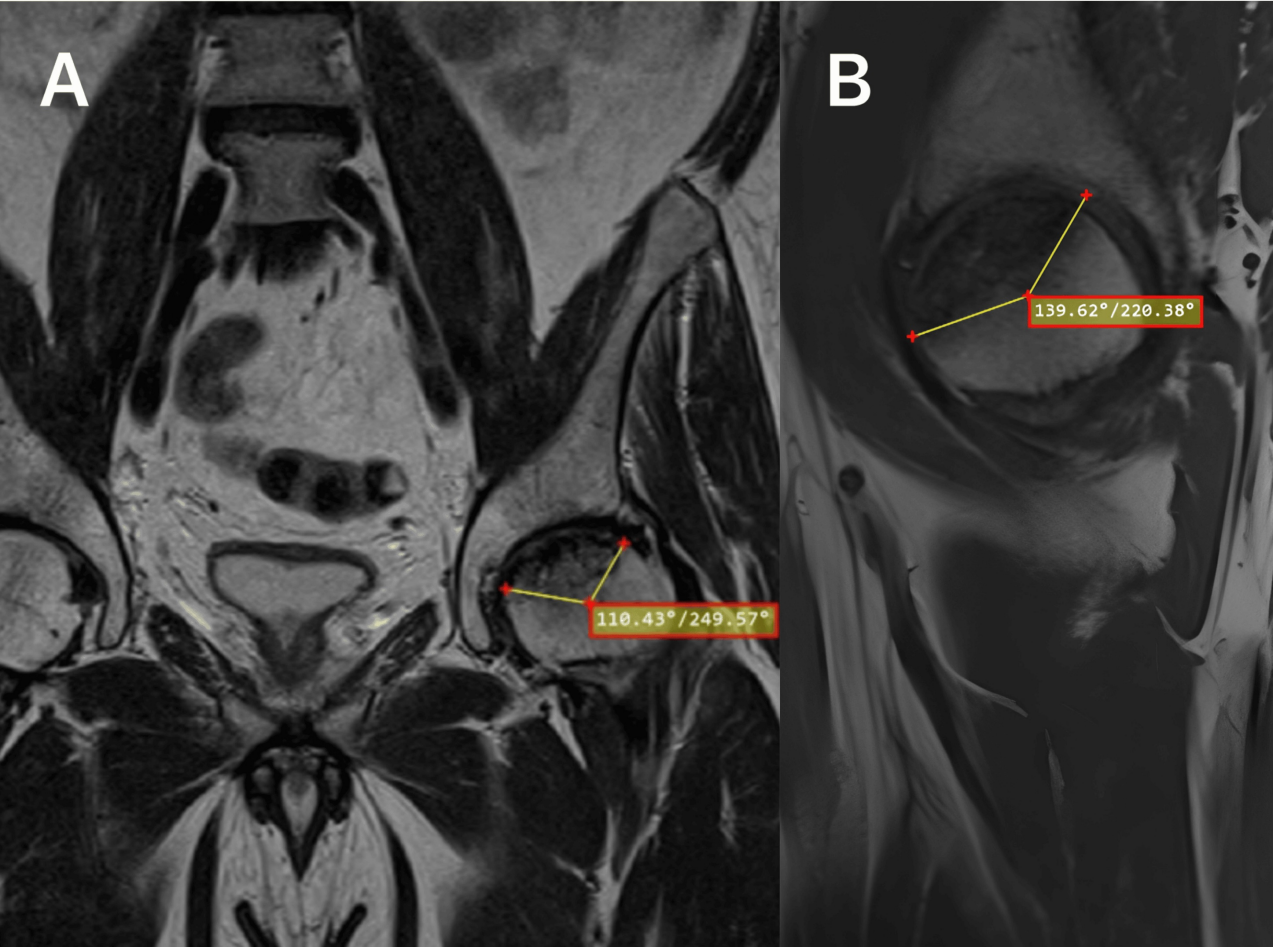
An example of the Cherian method, which estimates lesion size using angles (yellow lines) measured on MRI images showing maximum lesion size in (A) coronal and (B) sagittal slices

The modified necrotic index was calculated using the same formula but measured in the sagittal (angle A) and coronal (angle B) images showing the maximum lesion size, as described by Cherian et al. (Figure 2) [8].

MRI protocol

Patients were imaged in a supine position using a 1.5 Magnetom Avanto MRI scanner (Siemens Healthineers, Erlangen, DEU) with an 18-channel total imaging matrix (TIM) + daily optimization throughput (DOT) system. To ensure precise angular measurements, images were magnified so that the femoral head diameter measured 2.5 cm to 3.5 cm on a workstation.

Measurement protocol

Angular measurements were performed by two senior radiologists and one senior orthopedic surgeon. Each rater performed measurements on two separate occasions, and the mean values were used for final analysis.

Harris hip score

The HHS was evaluated by a resident not involved in the study. The score comprises of four domains: pain (44 points), function (47 points), range of motion (5 points), and absence of deformity (4 points), with a maximum score of 100. Scores were categorized as excellent (90-100), good (80-89), fair (70-79), and poor (<70).

Statistical analysis

The statistical analysis was performed using SPSS Statistics version 24. (IBM Corp., Armonk, NY, USA). Quantitative variables were expressed as the mean± the standard deviation, whereas qualitative variables were described as frequencies and percentages. Inter-rater reliability was assessed using the intraclass correlation coefficient (ICC). Variance and 95% confidence intervals (CI) for variance were estimated based on a normal distribution. Levene’s test for homogeneity of variance was used to compare variance across methods, serving as a measure of precision. A p-value < 0.05 was considered statistically significant.

## Results

Demographic characteristics

The mean age of the study participants was 40.82 ± 14.24 years, with a male-to-female ratio of two:one. The left and right hips were affected in 45 (51.14%) and 43 (48.86%) of the subjects, respectively. The mean duration of pain was 8.02 ± 5.99 months (range: one to 24 months). The most common comorbidities were obesity in 10 patients (18.5%) and diabetes mellitus in eight patients (14.8%). A definitive history of steroid use for more than three weeks was reported in 10 patients (18.5%), whereas seven patients (12.9%) had a history of COVID-19 infection. The majority of patients, i.e., 22 (40.7%), fell into the poor HHS category, followed by 12 (22.2%) in the fair category, 13 (24.1%) in the good category, and seven (13%) in the excellent category. The mean necrotic index was 25.44±17.52 %, while the mean modified necrotic index was 33.53±20.06 %.

ICC for osteonecrotic lesion assessment

The ICC for the modified necrotic index was 0.81 (95% CI: 0.61-0.913, p<0.05), whereas the ICC for the original necrotic index was 0.69 (95% CI: 0.127-0.747, p<0.05) (Table 1). Levene’s test for homogeneity of variance indicated unequal variance across the two methods (p < 0.01). The variance and corresponding 95% CI were smaller for the modified necrotic index (237; 95% CI: 183-265) compared to the necrotic index (348; 95% CI: 220-1002), suggesting greater precision with the modified method (Table 2).

**Table 1:** The ICC for the methods used to assess osteonecrotic lesion size ICC: Interclass correlation coefficient

Measurement method	ICC	95% CI	p-value
Cherian method	0.81	0.61-0.913	<0.05
Koo and Kim method	0.69	0.127-0.747	<0.05

**Table 2:** Results of Levene’s test to quantify precision of measurements made using each method

Measurement method	Estimate of variance using Levene's test	95% CI	p-value
Cherian method	237	183-265	<0.01
Koo and Kim method	348	220-1002	

Correlation of functional score with necrotic indices

Both indices showed significant negative correlations with HHS. The modified necrotic index demonstrated a strong negative correlation (r = -0.940; p < 0.001), whereas the necrotic index showed a moderate negative correlation (r = -0.790; p < 0.001). Both indices significantly decreased with increasing HHS (p = 0.0023) (Table 3).

**Table 3:** Correlation of functional score with necrotic indices

Measuement method	Mean	SD	Pearson correlation coefficient	p-value
HHS	71.28	15.32	-0.790	<0.001
Koo & Kim method	25.44	17.42		
Cherian method	33.53	20.06	-0.940	<0.001

Interobserver and intraobserver reliability and interobserver agreement

The time interval between measurements ranged from one week to one month. For the necrotic index, ICC values were 0.84 (p < 0.005) for the first rater, 0.64 (p < 0.005) for the second rater, and 0.58 (p < 0.005) for the third rater. For the modified necrotic index, ICC values were 0.75 (p < 0.005) for the first rater, 0.71 (p < 0.005) for the second rater, and 0.76 (p < 0.005) for the third rater (Table 4).

**Table 4:** Interobserver reliability across the indices

Measurement method	Intraclass correlation coefficient for the first rater	Intraclass correlation coefficient for the second rater	Intraclass correlation coefficient for the third rater	p-value
Cherian method	0.75	0.71	0.76	<0.005
Koo and Kim method	0.84	0.64	0.58	<0.005

Intraobserver agreement

For the necrotic index, ICC values were 0.65 (p < 0.001) for the first observation and 0.59 (p < 0.001) for the second observation. For the modified necrotic index, ICC values were 0.68 (p < 0.001) for the first observation and 0.78 (p < 0.001) for the second observation (Table 5).

**Table 5:** Intraobserver reliability across the indices

Measurement method	Intraclass correlation coefficient for the first observation	Intraclass correlation coefficient for the second observation	p-value
Cherian method	0.68	0.78	<0.001
Koo and Kim method	0.65	0.59	<0.001

## Discussion

The main findings of this study indicate that both the Koo and Kim, and the Cherian methods are useful for measuring the size of femoral head necrosis in ONFH. However, the modified necrotic index, as described by Cherian et al. [8], was found to be more precise in characterizing lesion size compared to the original necrotic index described by Koo et al. [7]. This was evidenced by the smaller variance for the modified necrotic index (237; 95% CI: 183-265) compared to the original necrotic index (348; 95% CI: 220-1002). Both methods demonstrated good interobserver and intraobserver reliability. Additionally, both indices showed a negative correlation with HHS, with the modified necrotic index exhibiting a strong correlation (r = -0.940; p < 0.001) and the original necrotic index showing a moderate correlation (r = -0.790; p < 0.001).

The treatment of ONFH is primarily guided by radiological parameters. Although the management of pre-collapse stages remains a topic of research and debate among orthopedic surgeons, there is consensus that lesion size and location play a critical role in determining prognosis and treatment. This underscores the importance of accurately measuring lesion sizes [11,12]. Early attempts to quantify necrosis were based on radiographs, such as the method proposed by Kerboul et al. [6]. In 1984, Steinberg et al. [13] incorporated lesion size into the staging of ONFH. With the advent of MRI, early detection of ONFH became possible, allowing for timely interventions in pre-collapse stages and potentially improving outcomes. The Association Research Circulation Osseous (ARCO) classification, introduced in 1991, integrated MRI findings and divided ONFH into different stages (zero-four), with subclassifications for lesion size and location, further enhancing prognostic accuracy [14].

Koo et al. [7] introduced the concept of quantifying the extent of necrosis using mid-coronal and mid-sagittal MRI images in the weight-bearing region to predict femoral head collapse. This method was later modified by Cherian et al. [8], who focused on measuring the largest area of necrosis rather than relying solely on mid-coronal and mid-sagittal images. Although the Koo and Kim method emphasizes lesion location (critical for predicting collapse) and is simple to measure by using 2D images, the Cherian method quantifies the overall burden of necrosis, irrespective of location. Other methods, such as volumetric measurements [9], provide three-dimensional assessments but are technically challenging and require specialized software, limiting their routine use.

Cherian et al. [8] examined 39 hips in 25 patients and found that both the necrotic index and the modified necrotic index were reproducible and reliable for quantifying osteonecrosis, making them clinically useful for identifying hips at the greatest risk of collapse. Hindoyan et al. [15] compared the precision of volumetric measurements with the Kerboul, Koo and Kim, and Cherian methods in 24 patients with ONFH. They observed that volumetric measurements were more precise (ICC = 0.81) compared to the Kerboul (ICC = 0.94), Koo and Kim (ICC = 0.61), and Cherian (ICC = 0.49) methods, though angular measurements sometimes overestimated lesion size. However, their sample size was small. In our study, which included 88 hips, the Cherian method demonstrated greater precision (ICC = 0.81; variance = 237; 95% CI: 183-265) compared to the Koo and Kim method (ICC = 0.69; variance = 348; 95% CI: 220-1002).

Although the utility of measuring necrotic indices for understanding disease severity and prognosis is well-established, combining radiological and clinical scores may provide a more comprehensive guide for management. However, literature on this correlation is scarce. In a cross-sectional study of 140 patients with ONFH, Krishnamurthy et al. [16] assessed the correlation between the Kerboul angle on MRI and HHS and reported a strong negative correlation (r = -0.647; p < 0.05). In our study, the Cherian method showed a strong negative correlation with HHS (r = -0.940; p < 0.001), whereas the Koo and Kim method demonstrated a moderate negative correlation (r = -0.790; p < 0.001). These findings suggest that a large extent of necrosis is associated with significantly reduced functionality. However, direct comparisons are challenging because of differences in measurement methods (indices vs. angular measurements).

Interobserver and intraobserver variability are critical factors in assessing the reliability of measurement methods. Cherian et al. [8] found that both methods could be used by single or multiple raters with high reliability. Our findings align with these results. In contrast, Kim et al. [17] reported unacceptable reliability for the Koo and Kim method, though their study was limited by a small sample size (six hips).

Limitations

This study has several limitations. The sample size was relatively small, and no prior sample size estimation was performed, which may affect the statistical power of the findings. Measurements were conducted by only three observers on two separate occasions. In some cases, lesions were irregular, patchy, or large, making precise angle measurements challenging. Additionally, patients were not followed up to assess disease progression, which limits the ability to evaluate the predictive value of these indices over time.

## Conclusions

Both the original necrotic index and its modified version serve as dependable tools for assessing lesion size in ONFH, though the modified index offers enhanced accuracy. While both indices displayed notable negative correlation with the HHS, the modified index showed a stronger correlation compared to the moderate association seen with the original index. Additionally, each method demonstrated significant interobserver and intraobserver reliability, supporting their reproducibility in practical use. Future research should focus on combining these indices with advanced imaging technologies and biomarkers to create comprehensive staging frameworks. Such multidimensional approaches may improve risk evaluation and enable more individualized treatment strategies for patients.
